# Traumatic Brain Injury Promotes Neurogenesis and Oligodendrogenesis in Subcortical Brain Regions of Mice

**DOI:** 10.3390/cells14020092

**Published:** 2025-01-10

**Authors:** Olga Astakhova, Anna Ivanova, Ilia Komoltsev, Natalia Gulyaeva, Grigori Enikolopov, Alexander Lazutkin

**Affiliations:** 1 Institute of Higher Nervous Activity and Neurophysiology, Russian Academy of Science, Moscow 117485, Russia; olga020302@gmail.com (O.A.);; 2 Department of Human and Animal Physiology, Faculty of Biology, Lomonosov Moscow State University, Moscow 119234, Russia; 3 Institute for Advanced Brain Studies, Lomonosov Moscow State University, Moscow 119991, Russia; anivis33@gmail.com; 4 Moscow Research and Clinical Center for Neuropsychiatry, Moscow 115419, Russia; 5 Center for Developmental Genetics, Stony Brook University, Stony Brook, NY 11794, USA; 6 Department of Anesthesiology, Stony Brook University, Stony Brook, NY 11794, USA

**Keywords:** brain trauma, fluid percussion injury, cell proliferation, adult neurogenesis, oligonendrogenesis, gliosis, striatum, substantia nigra, thalamus, optic tract

## Abstract

Traumatic brain injury (TBI) is one of the major causes of severe neurological disorders and long-term dysfunction in the nervous system. Besides inducing neurodegeneration, TBI alters stem cell activity and neurogenesis within primary neurogenic niches. However, the fate of dividing cells in other brain regions remains unclear despite offering potential targets for therapeutic intervention. Here, we investigated cell division and differentiation in non-neurogenic brain regions during the acute and delayed phases of TBI-induced neurodegeneration. We subjected mice to lateral fluid percussion injury (LFPI) to model TBI and analyzed them 1 or 7 weeks later. To assess cellular proliferation and differentiation, we administered 5-ethinyl-2′-deoxyuridine (EdU) and determined the number and identity of dividing cells 2 h later using markers of neuronal precursors and astro-, micro-, and oligodendroglia. Our results demonstrated a significant proliferative response in several brain regions at one week post-injury that notably diminished by seven weeks, except in the optic tract. In addition to active astro- and microgliosis, we detected oligodendrogenesis in the striatum and optic tract. Furthermore, we observed trauma-induced neurogenesis in the striatum. These findings suggest that subcortical structures, particularly the striatum and optic tract, may possess a potential for self-repair through neuronal regeneration and axon remyelination.

## 1. Introduction

Traumatic brain injury (TBI) is a major global health concern, contributing significantly to mortality and disability worldwide, with millions of new cases reported annually. TBI results in a broad spectrum of neurological impairments, including cognitive deficits, motor dysfunction, and emotional and behavioral disorders, many of which are often permanent and severely affect quality of life [1]. The long-term consequences of TBI are primarily associated with progressive neurodegeneration and limited regenerative capacity within the central nervous system. These processes contribute to extensive cellular damage and neural circuit disruptions, often resulting in irreversible functional impairments [2]. However, emerging evidence suggests that certain brain regions retain regenerative potential, offering hope for novel therapeutic strategies aimed at enhancing recovery.

The pathophysiology of TBI encompasses two phases: primary (acute) and secondary (delayed) nerve tissue damage [3]. The acute phase involves disruption of the blood-brain barrier (BBB) and neuronal and glial cell death [4]. This triggers local inflammatory processes and subsequent delayed neurodegeneration (delayed phase). Consequently, head injury often leads to persistent somatic, behavioral, and cognitive disruptions and deficits [5]. Although intrinsic compensatory mechanisms within the brain support some recovery, these are generally insufficient for substantial repair. Therapeutic interventions are, therefore, necessary to enhance neuroregenerative processes. Current treatment strategies focus on mitigating cell death during the delayed phase of TBI and promoting neuronal regeneration in damaged brain regions [4].

Neuroregenerative processes involve several stages, including neural stem cells (NSCs) and their progeny division, survival, migration to the lesion site, differentiation, and integration of newly generated neurons into existing functional circuits [6,7]. Each stage represents a potential therapeutic target, but the long-term effects of targeting specific stages within the NSC differentiation cascade remain uncertain. The mammalian brain maintains the capacity for neurogenesis throughout life, driven by the activity of NSCs located in specific neurogenic niches such as the subventricular zone (SVZ) of the lateral ventricles and the subgranular zone (SGZ) of the dentate gyrus of the hippocampus (SGZ) [7,8,9,10]. Additionally, there is evidence for distributed pools of NSCs in several other brain structures, such as the striatum, cortex, hypothalamus, amygdala, and substantia nigra [11,12].

TBI profoundly affects neurogenesis within main neurogenic niches, altering changes in NSC maintenance, progenitor cell division and migration pathways, and differentiation [13,14,15,16,17]. Beyond these niches, brain injury induces a robust proliferative response associated with active gliosis in non-neurogenic regions [18,19,20,21,22,23,24,25]. These findings suggest that non-neurogenic regions may contribute to the repair of neural circuits and remyelination, highlighting their potential as therapeutic targets.

In this study, we aim to investigate the proliferative response and differentiation pathways of cells outside the primary neurogenic niches following TBI. Using a lateral fluid percussion injury (LFPI) model of TBI, we examine both the acute and delayed phases of the TBI-induced damage, focusing on cellular proliferation and on the differentiation of dividing cells into neuronal, astrocytic, microglial, and oligodendroglial lineages. Our study seeks to uncover the regenerative potential of the brain beyond traditional neurogenic zones, with implications for enhancing recovery after traumatic injury.

## 2. Materials and Methods

### 2.1. Animals

The experiment was performed on adult male heterozygous Nestin-GFP mice [26] maintained on C57BL/6J background aged 3.5–4 months on the day of surgery. Mice were housed in cages 36 × 21 × 13.5 cm in groups of 6–7 animals per cage under standard conditions with a 12 h/12 h light/dark cycle, with free access to food and water. All experiments were approved by the Ethical Commission of the Institute of Higher Nervous Activity & Neurophysiology, Russian Academy of Sciences (Protocol No. 2, 24 December 2017) and Stony Brook University IACUC. Every effort was made to minimize animal pain and distress.

### 2.2. Lateral Fluid Percussion Injury Model

The LFPI in rodents is believed to be a gold-standard TBI model with high validity in translational studies [27,28,29,30]. As compared to other popular TBI models such as control cortical impact (CCI) or weight drop, the LFPI induces a broader spread, diffuse trauma involving subcortical structures [31,32]. Also, compared to the CCI model, LFPI does not cause damage to the dura mater, which minimizes the effect of the surgery itself on the proliferative response that was the focus of our study [33]. Although the LFPI model’s limitations may include some variability, brain stem damage, and the lack of long-lasting coma [34], this TBI model is more appropriate for the study’s aim due to its higher clinical relevance.

Before surgery, the animals were randomly divided into 3 groups: “Intact Control” (IC)—non-operated animals from a home cage (*n* = 13), “Sham” (Sh)—operated mice without injury (*n* = 19), and “Trauma” (Tr)—the post-TBI group (*n* = 24). The surgery was performed under 1–2% isoflurane anesthesia. Mice were scalped using a midline incision. A 4 mm diameter trepanation aperture was created in the right parietal bone with an electric trepanner (Bregma = −2 mm, Lateral = 2 mm). A head of a Luer-type needle was attached to the aperture with cyanoacrylate glue and filled with sterile saline solution. Mice were disconnected from isoflurane anesthesia, and after regaining consciousness, the animals were placed in a rodent restraint bag. A head of the Luer-type needle was connected by a plastic tube to the LFPI device (Fluid Percussion Device with the PC-Based Pressure Measurement Unit, Model FP302, Richmond, VA, USA). Using the LFPI device, the mice in the “Trauma” group were injured by a force of about 2 atm. Then, the animals were disconnected from the device, and the duration of the righting reflex recovery (time of spontaneous turning from the left and right sides to the paws) was recorded. After wound suturing under isoflurane anesthesia, the animals were returned to their home cages. Sham mice were subjected to all procedures except injury by the LFPI device. The duration of the craniotomy was about 30 min. The duration of wound suturing was about 15 min.

### 2.3. Cell Labeling

To investigate the proliferative activity of the injured brain at different time points after TBI, each of the three groups was randomly divided into two subgroups. To label dividing cells in the brain, animals were injected with 5-ethynyl-2′-deoxyuridine (EdU, 40 mg/kg, 30540, Lumiprobe, Moscow, Russia) i.p. [35,36] 1 or 7 weeks after injury. The selected time points correspond to the acute phase of the response to TBI (1 week) when a peak proliferative response is observed, and the delayed phase of the response (7 weeks), when a decrease in the inflammatory response is observed, as based on current literature data [19,37]. Two hours after EdU injection, mice were deeply anesthetized with chloral hydrate (1500 mg/kg, C8383, Sigma-Aldrich, St. Louis, MO, USA) and perfused with 30 mL of phosphate-buffered saline (PBS, pH = 7.4) (18912014, Thermo Fisher Scientific, Waltham, MA, USA), and 30 mL of 4% paraformaldehyde (PFA) (158127, Sigma-Aldrich, St. Louis, MO, USA). The brains were postfixed in 4% PFA overnight at 4 °C and transferred to PBS for storage at +4 °C.

### 2.4. Staining

The right injured hemispheres were sliced into sagittal 50 μm sections using 7000smz-2 vibratome (Campden Instruments, Loughborough, UK). The sections were collected in 6 wells of 24-well tissue culture plates with a 300 μm distance between sections in each well [38]. Sets of sections from one well were used for one staining and cell counting. The sections were kept in PBS at 4 °C or in cryoprotectant (2 volumes of PBS, 1 volume of ethylene glycol, and 1 volume of glycerin, the last two from Sigma-Aldrich, St. Louis, MO, USA) at −20 °C until staining.

For each group of mice, six sets of brain sections were selected for further staining. Sections were stained in 24-well tissue culture plates: EdU^+^ cells were detected by click-histochemistry, and other cell markers were detected by immunohistochemistry. Staining of sections was performed with fluorescent cell markers with the following excitation wavelengths: 405 nm—Hoechst 33342, 488 nm—GFP, 568 nm—EdU, 647 nm—GFAP, Iba1, Olig2, and DCX. All procedures due to staining were performed with gentle shaking.

*DCX staining.* Sections for DCX-staining were rinsed from cryoprotectant three times with PBS at room temperature (RT). Then, sections were permeabilized with 2% Triton X-100 (Sigma-Aldrich, St. Louis, MO, USA) and 5% normal goat serum (NGS) (ab7481, Abcam, Cambridge, UK) in PBS for 1 h at RT. After three rinses with 0.2% Triton X-100 in PBS, the sections were incubated with guinea pig anti-DCX primary antibodies (1:5000, AB2253, Millipore, St. Louis, MO, USA) in PBS containing 0.2% Triton X-100 and 5% NGS overnight at RT. After three washes with 0.2% Triton X-100 in PBS, the sections were stained with goat anti-guinea pig AlexaFluor 647 antibody (1:500, A21450, Invitrogen, Waltham, MA, USA), 0.2% Triton X-100 and 5% NGS in PBS for 2 h in the dark at RT. The negative control of secondary antibodies was performed by replacing the primary antibody with the equivalent amount of normal goat serum. Then, sections were washed three times with 0.2% Triton X-100 in PBS and three times with PBS, and after that, sections were mounted on glass slides using Fluorescence Mounting Medium (S3023, Dako, St. Clara, CA, USA). The slides were dried horizontally overnight at RT and stored at +4 °C until imaging.

*EdU staining.* EdU^+^ cells were detected with three sets of cell markers: Staining 1—Hoechst 33342, GFP, EdU, GFAP; Staining 2—Hoechst 33342, EdU, Iba1; Staining 3—Hoechst 33342, EdU, Olig2. Sections for EdU-staining were washed from cryoprotectant and permeabilized as described previously. After three rinses with 0.2% Triton X-100 in PBS, the sections were incubated with primary antibodies overnight at RT. Primary antibodies were diluted in PBS with 0.2% Triton X-100 and 5% NGS. The following antibodies were used: chicken anti-GFP (1:500, GFP-1020, Aves, Davis, CA, USA) and rabbit anti-GFAP (1:250, 180063, Invitrogen, Waltham, MA, USA) for the first staining; rabbit anti-Iba1 (1:500, 019-19741, Wako, Osaka, Japan) for the second staining; rabbit anti-Olig2 (1:1000, AB9610, Millipore, St. Louis, MO, USA) for the third staining. After three rinses with 0.2% Triton X-100 in PBS, the sections were incubated with goat anti-chicken Alexa Fluor 488 (a11039, Molecular Probes, Eugene, OR, USA) and goat anti-rabbit Alexa Fluor 647 (A21244, Molecular Probes, Eugene, OR, USA) for the first staining; with goat anti-rabbit Alexa Fluor 647 for the second and third staining in PBS containing 0.2% Triton X-100, 5% NGS and Hoechst 33342 (1 μg/mL, H3570, Invitrogen, Waltham, MA, USA) for 2 h in the dark at RT. The negative control of secondary antibodies was performed by replacing the primary antibody with the equivalent amount of normal goat serum. A click reaction was performed with 0.2% Triton X-100, 120 mM sodium ascorbate (Sigma-Aldrich, St. Louis, MO, USA), 100 mM CuSO_4_ (Sigma-Aldrich, St. Louis, MO, USA), and 1 mM AF568 azide (A6820, Lumiprobe, Moscow, Russia) in PBS for 20 min at RT in the dark. The click reaction was stopped by incubation with 0.1M EDTA (pH = 8) (Invitrogen, Waltham, MA, USA). The sections were rinsed three times with 0.2% Triton X-100 in PBS and three times with PBS. Sections were mounted on glass slides using a Fluorescence Mounting Medium (S3023, Dako, St. Clara, CA, USA). The slides were dried horizontally overnight at RT and stored at +4 °C until imaging.

### 2.5. Image Analysis

For the analysis of the density of EdU^+^ cells in the injured brain at 1 and 7 weeks after TBI, sections were imaged using a fluorescent microscope Leica DM6 B (Leica Microsystems, Wetzlar, Germany) with a 10× objective (NA 0.32). The density of EdU-positive cells was counted using Imaris 7.6.4 software (RRID: SCR_007370, Bitplane, Belfast, UK). Two levels of the brain were analyzed: medial (Lateral = 0.84 to 1.44 mm) and lateral (Lateral = 2.64 to 3.36 mm). Both levels were chosen to count the maximum amount of different brain structures at the same distance from the center of the injured area. Cell counting on sections at the region of injury was not performed due to substantial damage to brain structures at this level. At the medial level, the density of EdU^+^ cells was analyzed in the frontal associative and secondary motor cortexes, striatum, thalamus, substantia nigra, and tectum. At the lateral level—in the somatosensory, insular, piriform, and entorhinal cortexes, as well as in the striatum and amygdala. Three slices from each level were selected for counting. Cell density was calculated as the cell number ratio per structure to the structure area, averaged for 3 slices. In addition, the same protocol was used to count cells in the optic tract (Lateral = 1.20 to 2.40 mm), which is located beyond the levels described previously. At the medial level, the cerebellum, brainstem, and reticular formation were not included in the analysis, as it was not possible to collect three slices containing these structures for all animals due to cutting features. In addition, the visual and somatosensory cortexes at the medial level and the visual cortex at the lateral level were not included in the analysis because of the location of these structures at the injury area and, as a result, their damage. Moreover, the rostral migratory stream was excluded from the count because of the high density of EdU^+^ cells in these areas and the impossibility of the density estimation by the chosen method.

For analysis of DCX^+^ and EdU^+^ cells colocalized with different markers, we selected several structures: substantia nigra, striatum, optic tract, and thalamic nuclei—laterodorsal, lateral posterior, and ventral—that were located on at least three sagittal slices. Structures were selected based on the highest cell density of EdU^+^ cells calculated as described above. The phenotype of EdU^+^ cells was analyzed with the following sets of cell markers: Nestin-GFP and GFP were used to study astrogliosis and neurogenesis; Nestin-GFP and Olig2 were used to study oligodendrogenesis; Nestin-GFP and Iba1 were used to study microgliosis. Z-stack images of sections were obtained using a fluorescent microscope Leica DM6 B with a 20× objective (NA 0.55) using the Instant Computational Clearing (ICC) post-processing algorithm. The number of cells in each structure was counted on 3 slices and averaged for each structure using Imaris 7.6.4 software. The total number of GFAP- and Iba1-labeled cells was analyzed with ImagePro Plus v.3.0 software (RRID: SCR_016879, Media Cybernetics, Rockville, MD, USA). One slice was selected for each brain of each structure, and the density of GFAP- or Iba1-labeled cells was counted using a semiautomated method.

### 2.6. Statistical Analysis

Statistical analysis and graph plotting were performed using GraphPad Prism version 9.5.1 (GraphPad Software, San Diego, CA, USA). The ROUT method (Q = 1%) was used to identify outliers. The distribution of data in each set of experiments was tested for normality using the Shapiro-Wilk test. The Wilcoxon test was applied to compare the duration of righting reflex recovery. One sample t-test was conducted to analyze the force of LFPI. The significance between the three groups of the same period (“Intact Control”, “Sham”, and “Trauma”) was determined with an ordinary one-way ANOVA test, followed by Tukey’s post hoc analysis. The difference between the three experimental groups at two time points was compared using ordinary two-way ANOVA and Tukey’s multiple comparisons test. Statistical differences were considered significant at a *p* value less than 0.05. All data sets were presented in the graphs as a mean ±SEM.

## 3. Results

### 3.1. Traumatic Brain Injury Induced by Lateral Fluid Percussion Injury Model

**Figure 1:**
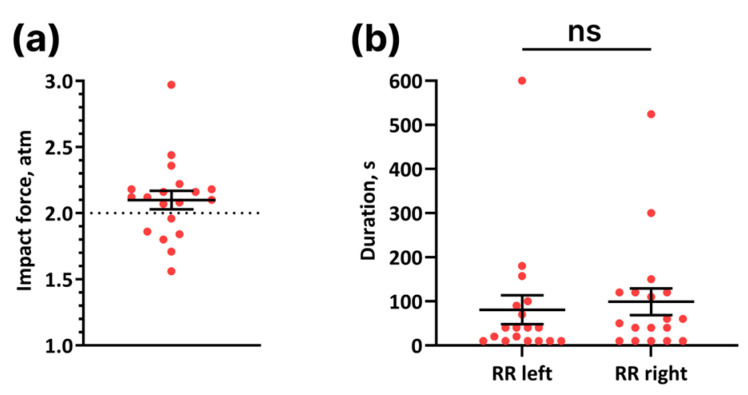
Lateral fluid percussion injury model. (**a**) Experimental impact force in mice with traumatic brain injury with a target force of 2 atm (*n* = 19). One-sample *t*-test, *p* = 0.17. (**b**) Left and right righting reflex recovery (RR) (*n* = 18). Wilcoxon test, ns—*p* = 0.5. (**a,b**) Data are presented as mean ± SEM.

The experimental impact force in all mice subjected to LFPI averaged 2.1 ± 0.1 atm (*n* = 24), which was not significantly different from the target impact force of 2 atm (Figure 1a; *p* = 0.17, one sample *t*-test). The lethality rate among the LFPI mice was 20.8% (*n* = 5). The recovery time from anesthesia varied between 3 to 44 min. To account for the variability in anesthesia recovery times, the injury was administered after the animals regained the vestibulospinal reflex (righting reflex). The recovery time for the righting reflex after LFPI was 99 ± 30 s (right side/ipsilateral) and 81 ± 33 s (left side/contralateral) (Figure 1b; *p* = 0.5, Wilcoxon test). Thus, in terms of impact force and death rate, the severity of TBI in our paper corresponded to moderate [29].

### 3.2. LFPI Induced Increases in Cell Divisions in the Injured Hemisphere One, but Not Seven Weeks Post-Injury

**Figure 2:**
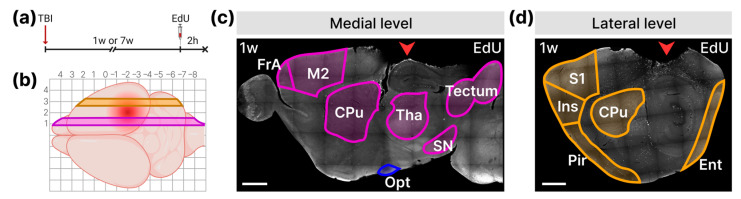
Principle of cell division analysis in the brain after TBI. (**a**) Scheme of experiment. At 1 or 7 weeks after LFPI, mice were injected with EdU; brains were collected 2 h after injection. (**b**) The levels for analyzing the density of EdU-labeled cells in the injured hemisphere: lateral level (orange), medial level (purple). The red spot indicates the lesion site. (**c**) Analyzed structures at the medial level. The purple lines outline the analyzed structures: M2—secondary motor cortex; FrA—frontal association cortex; CPu—striatum; Tha—thalamus; SN—substantia nigra; Tectum. The blue line circles the optic tract (Opt) that partially enters the medial level. (**d**) Analyzed structures at the lateral level. The orange lines outline the analyzed structures: S1—somatosensory cortex, Ins—insular cortex; Pir—piriform cortex; Ent—entorhinal cortex; CPu—striatum. (**c**,**d**) Representative pictures show the distribution of EdU^+^ cells (white) at both levels 1 week (1 w) after TBI. Red arrowheads indicate the lesion site. The scale bar is 1000 µm.

To identify non-neurogenic brain regions exhibiting the most significant proliferative response, we injected the dividing cell marker EdU into mice 1 and 7 weeks after LFPI and collected the brain samples 2 h later (*n* = 6 for each group) (Figure 2a). The density of EdU-labeled cells was analyzed at two levels equidistant from the injured site: medial and lateral (Figure 2b–d).

**Figure 3:**
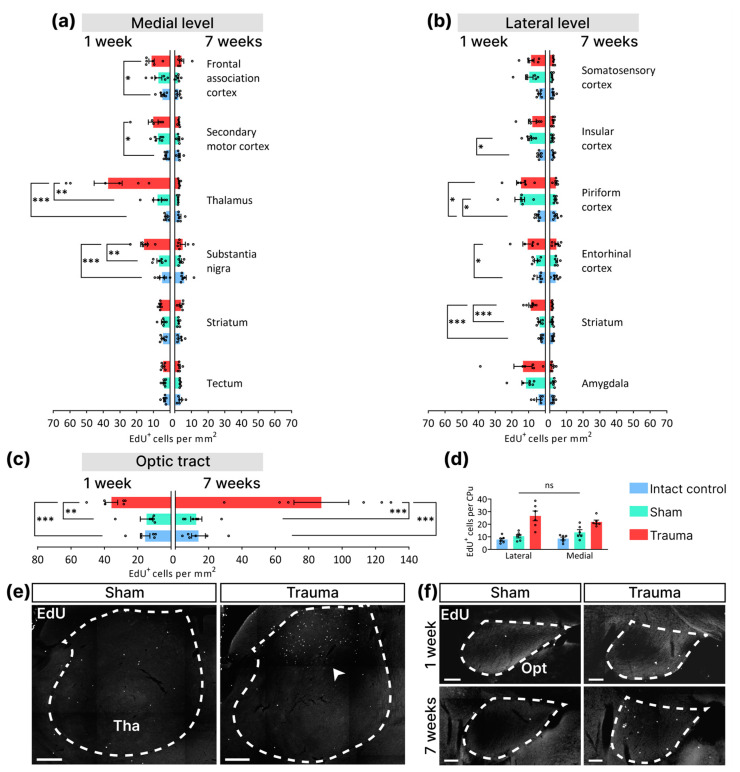
Traumatic brain injury leads to an extensive increase in cell divisions in the injured hemisphere 1 week after injury. (**a**) EdU^+^ cell density at the medial level 1 and 7 weeks after LFPI. (**b**) EdU^+^ cell density at the lateral level 1 and 7 weeks after LFPI. (**c**) EdU^+^ cell density in the optic tract 1 and 7 weeks after LFPI. (**d**) Comparison of EdU^+^ cell numbers in the striatum at both levels 1 week after TBI. Two-way ANOVA. Factor “Level”: ns—*p* = 0.8543; factor “Impact”: *p* < 0.0001; interaction: *p* = 0.1713 (*n* = 6 for each group). (**e**) Representative images of EdU^+^ (white) cells in the thalamus (Tha) after 1 week in the “Sham” and “Trauma” groups. The dotted line indicates the boundaries of the thalamus. The white arrowhead indicates an increase in EdU^+^ cells in the dorsal part of the striatum after TBI. The scale bar is 300 µm. (**f**) Representative images of EdU^+^ (white) cells in the optic tract (Opt) at 1 and 7 weeks after TBI in “Sham” and “Trauma” groups. The scale bar is 100 µm. The dotted line indicates the boundaries of the optic tract. (**a**–**c**) One-way ANOVA, Tukey’s post-hoc test. *—*p* < 0.5, **—*p* < 0.01, ***—*p* < 0.001. Data are presented as mean ± SEM (*n* = 6 for each group).

One week post-impact, mice of the “Trauma” group displayed a higher density of EdU^+^ cells than animals of the “Intact Control” and “Sham” control groups. This increase was observed in the thalamus (IC: 2 ± 0.5, Sh: 8 ± 2.4, Tr: 37 ± 8.4, *p* = 0.0004, one-way ANOVA, Tukey’s post-hoc test), substantia nigra (IC: 5 ± 0.9, Sh: 7 ± 1.4, Tr: 16 ± 1.3, *p* = 0.0005, one-way ANOVA, Tukey’s post-hoc test) at the medial level (Figure 3a); in the striatum at the lateral level (IC: 2 ± 0.4, Sh: 3 ± 0.6, Tr: 9 ± 1.2, *p* = 0.0001, one-way ANOVA, Tukey’s post-hoc test) (Figure 3b); and in the optic tract (IC: 16 ± 2.9, Sh: 15 ± 3.8, Tr: 36 ± 3.8, *p* = 0.0009, one-way ANOVA, Tukey’s post-hoc test) (Figure 3c). Remarkably, the thalamus exhibited the most significant rise in the number of EdU^+^ cells (Figure 3a), predominantly localized in the dorsal region of the structure (Figure 3e). In injured animals, an increase in EdU^+^ cells compared to the “Intact Control” group was observed in the frontal associative (IC: 5 ± 1.0, Sh: 7 ± 2.1, Tr: 11 ± 1.6, *p* = 0.0383, one-way ANOVA, Tukey’s post-hoc test) and secondary motor (IC: 2 ± 0.4, Sh: 7 ± 1.2, Tr: 10 ± 2.9, *p* = 0.0305, one-way ANOVA, Tukey’s post-hoc test) cortexes at the medial level (Figure 3a), and in the piriform (IC: 5 ± 0.5, Sh: 15 ± 2.9, Tr: 15 ± 2.7, *p* = 0.0074, one-way ANOVA, Tukey’s post-hoc test) and entorhinal (IC: 4 ± 0.9, Sh: 6 ± 0.9, Tr: 11 ± 2.9, *p* = 0.0426, one-way ANOVA, Tukey’s post-hoc test) cortexes at the lateral level (Figure 3b), In addition, differences were found between “Sham” and “Intact Control” groups in the insular (IC: 4 ± 0.5, Sh: 9 ± 1.2, Tr: 8 ± 2.3, *p* = 0.0464, one-way ANOVA, Tukey’s post-hoc test) and piriform cortexes at the lateral level (Figure 3b). No statistically significant differences were found in the striatum (IC: 4 ± 0.7, Sh: 4 ± 0.8, Tr: 6 ± 0.3, *p* = 0.1189, one-way ANOVA, Tukey’s post-hoc test), and tectum (IC: 3 ± 0.5, Sh: 4 ± 0.4, Tr: 4 ± 0.5, *p* = 0.5779, one-way ANOVA, Tukey’s post-hoc test) at the medial level (Figure 3a), and in the somatosensory cortex (IC: 4 ± 0.5, Sh: 10 ± 2.2, Tr: 9 ± 1.8, *p* = 0.0527, one-way ANOVA, Tukey’s post-hoc test) and amygdala (IC: 4 ± 1.1, Sh: 12 ± 2.5, Tr: 13 ± 5.4, *p* = 0.1727, one-way ANOVA, Tukey’s post-hoc test) at the lateral level (Figure 3b). The lack of differences between groups in the striatum at the medial level can be attributed to an uneven distribution of labeled cells in the dorsoventral direction and its larger slice area than at the lateral level. To minimize this effect, we compared the number of EdU-positive cells instead of the density in the striatum at both levels. Two-way ANOVA showed a significant difference in the factor “Impact” and not the factor “Level” (Factor “Level”: *p* = 0.8543; factor “Impact”: *p* < 0.0001; interaction: *p* = 0.1713) (Figure 3d). In addition, Tukey’s multiple comparisons test revealed a significant increase in the number of EdU-labeled cells at both levels in the injured striatum compared to the two control groups, with no differences observed between levels of EdU^+^ cells within the “Trauma” group.

Seven weeks after LFPI, no changes in the density of EdU-labeled cells were detected in any of the analyzed structures at both the medial and lateral levels (Figure 3a,b). The optic tract was the only structure that showed a significantly extensive increase in the density of EdU^+^ cells 7 weeks after injury compared to the two control groups (IC: 14 ± 4.1, Sh: 13 ± 3.4, Tr: 88 ± 16.4, *p* < 0.0001, one-way ANOVA, Tukey’s post-hoc test) (Figure 3c,f).

These data indicate that LFPI-induced cell divisions in most of the investigated structures of the injured hemisphere 1 week after the impact. Trauma, in contrast to sham surgery, not only resulted in an increased density of dividing cells in various cortical regions but also elicited a proliferative response in subcortical structures, including the striatum, substantia nigra, thalamus, and optic tract. Seven weeks post-injury, nearly all brain structures showed a reduction in proliferative activity to control levels, except for the optic tract.

### 3.3. Traumatic Brain Injury Altered the Differentiation Pathways of Newly Formed Cells

We determined that LFPI induced a solid proliferative response in subcortical structures, including the striatum, substantia nigra, thalamus, and optic tract. To establish the initial stages of dividing cell differentiation, we identified the phenotype of EdU-labeled cells one week post-impact in the striatum, thalamus, and substantia nigra, and seven weeks post-impact in the optic tract. Phenotyping was performed with the following markers: Nestin-GFP for stem and progenitor cells, GFAP for astrocytes and radial glia cells, Olig2 for oligodendrocytes and their precursors, Iba1 for microglia. Combining these markers allowed for a more precise determination of the phenotype of dividing cells [10,33,39,40]. In the SGZ, combinations of Nestin-GFP and GFAP markers facilitate the detection of the following cell types: EdU^+^/Nestin-GFP^+^/GFAP^+^—radial glia-like cells (RGLs); EdU^+^/Nestin-GFP^−^/GFAP^+^—astrocyte precursors; EdU^+^/Nestin-GFP^+^/GFAP^−^—amplifying neuronal precursors [10]. Thus, the neurogenesis and astrogliosis in the hippocampus can be characterized by Nestin-GFP and GFAP markers. In addition, different combinations of Olig2 and Nestin-GFP markers characterize different stages of differentiation of newly formed oligodendrocytes: EdU^+^/Nestin-GFP^+^/Olig2^+^—oligodendrocyte precursor cells (OPCs); EdU^+^/Nestin-GFP^−^/Olig2^+^—immature and mature oligodendrocytes [41]. Therefore, guided by hippocampal phenotyping data, we determined whether these marker combinations occur in other brain regions during post-injury proliferation.

#### 3.3.1. Striatum

**Figure 4:**
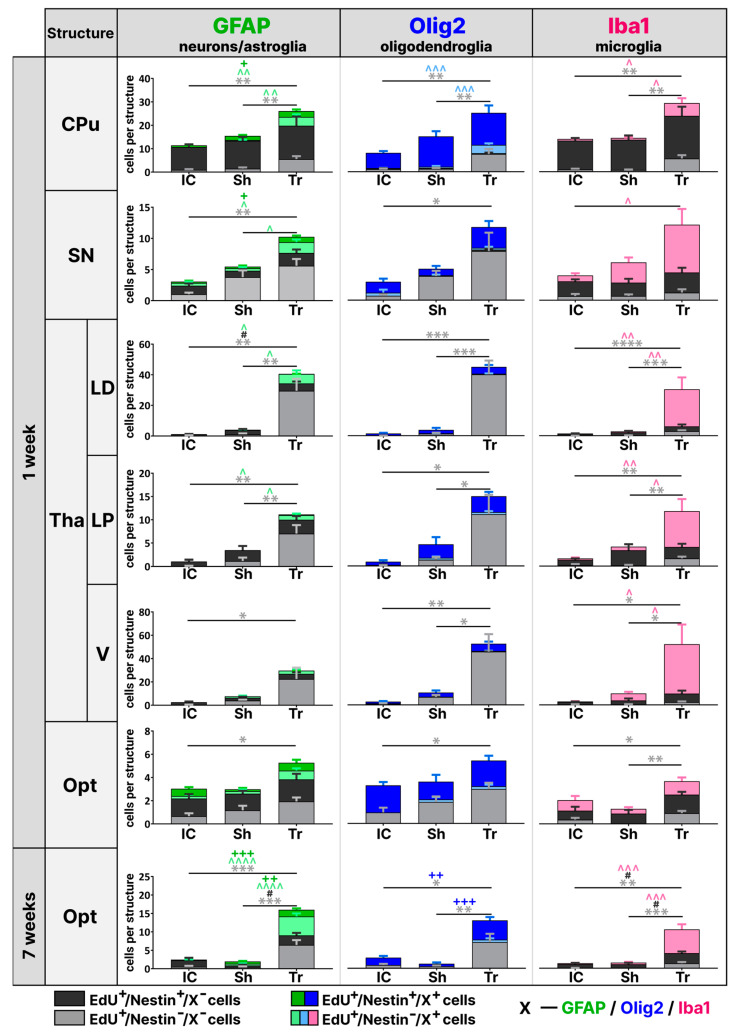
LFPI changed the differentiation pathways of newly formed cells in subcortical brain structures. The figure shows the results of phenotyping EdU^+^ cells with either the marker GFAP (green column), Olig2 (blue column), or Iba1 (pink column) combined with Nestin-GFP. EdU^+^/Nestin^−^/X^−^ cells—light gray column; EdU^+^/Nestin^+^/X^−^ cells—dark gray column; EdU^+^/Nestin^−^/X^−^ cells—light green/blue/pink column; EdU^+^/Nestin^+^/X^−^ cells—dark green/blue column; X—one of the specific markers GFAP, Olig2 or Iba1. Phenotyping was performed in the striatum, substantia nigra, and thalamic nuclei at 1 week after LFPI and in the optic tract at 1 and 7 weeks after injury. Abbreviations: CPu—striatum, SN—substantia nigra, Tha—thalamus, LD—laterodorsal thalamic nucleus, LP—lateral posterior thalamic nucleus, V—ventral thalamic nucleus, Opt—optic tract, IC – Intact Control, Sh – Sham, Tr - Trauma. One-way ANOVA, Tukey’s post-hoc test. *—*p* < 0.5, **—*p* < 0.01, ***—*p* < 0.001, ****—*p* < 0.0001, EdU^+^/Nestin^−^/X^−^ cell differences; #—*p* < 0.5, EdU^+^/Nestin^+^/X^−^ cell differences; ^—*p* < 0.5, ^^—*p* < 0.01, ^^^—*p* < 0.001, ^^^^—*p* < 0.0001, EdU^+^/Nestin^−^/X^+^ cell differences; +—*p* < 0.5, ++—*p* < 0.01, +++—*p* < 0.001, EdU^+^/Nestin^+^/X^+^ cell differences. Data are presented as mean ± SEM (*n* = 6 for each group).

**Figure 5:**
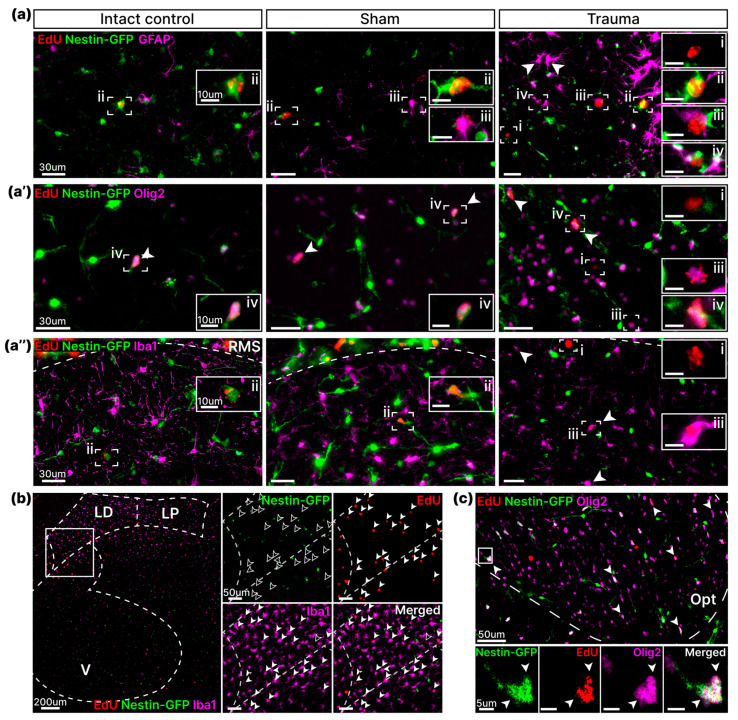
Traumatic brain injury induced irregular gliosis in the brain. (**a**,**a′**,**a″**) Representative images show colocalization of EdU-labeled cells with a combination of GFAP (**a**) or Olig2 (**a′**) or Iba1 (**a″**) and Nestin-GFP markers in the striatum 1 week after LFPI. Solid box: cell in the dashed box at higher magnification. i—EdU^+^/Nestin^−^/X^−^ cells; ii—EdU^+^/Nestin^+^/X^−^ cells; iii—EdU^+^/Nestin^−^/X^+^ cells; iv—EdU^+^/Nestin^+^/X^+^ cells; where X is one of the specific markers: GFAP, Olig2, or Iba1. (**a**) White arrowheads indicate hypertrophied GFAP-positive cells. (**a′**) White arrowheads indicate EdU^+^/Nestin^+^ cells, all of which colocalize with Olig2. (**a″**) White arrowheads indicate EdU^+^/Nestin^−^/Iba1^+^ cells located near the rostral migratory stream (RMS). (**b**) Representative images show active irregular thalamic microgliosis seven weeks post-injury. The laterodorsal nucleus (LD), lateral posterior nucleus (LP), and ventral nucleus (V) of the thalamus are indicated by the dotted line. The white box is bounded by the fragment, represented on the right at higher magnification in three separate channels. Filled arrowheads indicate the presence of marker expression in the cell. Empty arrowheads indicate the absence of marker expression in the cell. (**c**) EdU^+^/Nestin-GFP^+^/Olig2^+^ OPCs in the optic tract 7 weeks after LFPI. The dashed line indicates the optic tract (Opt). White arrowheads point to EdU^+^/Nestin^−^GFP^+^/Olig2^+^ cells. The white box bounds the cell shown below at higher magnification in three separate channels.

First, we performed phenotyping of EdU-labeled cells in the striatum. We found that the majority of dividing cells in all groups colocalized with the Nestin-GFP marker (Figure 4, light gray). In addition, most of the EdU^+^/Nestin-GFP^+^ cells co-labeled with the Olig2 marker (Figure 4 dark blue; Figure 5a′). Furthermore, no EdU^+^/Nestin-GFP^+^ cell co-localized with Iba1 in the striatum and other structures described below (Figure 4, absence of dark pink; Figure 5a″). These data indicate that most of the dividing cells in the striatum were OPCs. By phenotyping dividing cells with GFAP, we found that LFPI resulted in an increase in the number of EdU^+^/Nestin-GFP^−^/GFAP^+^ cells compared to the two control groups (IC: 0 ± 0, Sh: 0 ± 0.2, Tr: 4 ± 1.2, *p* = 0.0027, one-way ANOVA, Tukey’s post-hoc test) and EdU^+^/Nestin-GFP^+^/GFAP^+^ cells (IC: 1 ± 0.6, Sh: 2 ± 1.0, Tr: 3 ± 1.7, *p* = 0.046, one-way ANOVA, Tukey’s post-hoc test) compared to the “Intact Control” group (Figure 4 and Figure 5a). Moreover, there was a change in the morphology of GFAP^+^ cells, which had a hypertrophied cell body and thickened branches (Figure 5a). We also observed an increase in the number of EdU^+^ cells not labeled with Nestin-GFP and GFAP (IC: 1 ± 0.3, Sh: 1 ± 0.5, Tr: 6 ± 1.3, *p* = 0.0022, one-way ANOVA, Tukey’s post-hoc test) (Figure 4 and Figure 5a), which was achieved by increasing the number of EdU^+^/Nestin-GFP^−^/Olig2^+^ (IC: 0 ± 0.1, Sh: 1 ± 0.3, Tr: 4 ± 0.7, *p* <0.0001, one-way ANOVA, Tukey’s post-hoc test) (Figure 4 and Figure 5a′) and EdU^+^/Nestin-GFP^−^/Iba1^+^ cells (IC: 1 ± 0.3, Sh: 1 ± 0.3, Tr: 6 ± 2.1, *p* = 0.0217, one-way ANOVA, Tukey’s post hoc test) (Figure 4 and Figure 5a″). Moreover, active microgliosis occurred in the region closest to the site of impact—near the RMS (Figure 5a″).

#### 3.3.2. Substantia Nigra

Phenotyping of dividing cells in the substantia nigra showed that the increase in EdU-labeled cells following TBI was due to the active division of EdU^+^/Nestin-GFP^−^/Iba1^+^-labeled microglia (IC: 1 ± 0.3, Sh: 3 ± 0.8, Tr: 8 ± 2.6, *p* = 0.0234, one-way ANOVA, Tukey’s post-hoc test) (Figure 4). In addition to active microglia, we also found an increased number of EdU^+^/Nestin-GFP^−^/GFAP^+^ astrocytes compared with the two control groups (IC: 0 ± 0.2, Sh: 0 ± 0.2, Tr: 2 ± 0.4, *p* = 0.0123, one-way ANOVA, Tukey’s post-hoc test), and an increased number of EdU^+^/Nestin-GFP^+^/GFAP^+^ astrocytes with morphology similar to activated astrocytes in the striatum (IC: 0 ± 0.1, Sh: 0 ± 0.2, Tr: 1 ± 0.2, *p* = 0.0335, one-way ANOVA, Tukey’s post-hoc test) (Figure 4). In addition, in contrast to striatum, the substantia nigra displayed no changes in the number of either EdU^+^/Nestin-GFP^−^/Olig2^+^ + (IC: 1 ± 0.5, Sh: 0 ± 0.1, Tr: 0 ± 0.2, *p* = 0.6866, one-way ANOVA, Tukey’s post-hoc test) or EdU^+^/Nestin-GFP^+^/Olig2^+^ (IC: 2 ± 0.5, Sh: 1 ± 0.5, Tr: 3 ± 1.0, *p* = 0.0856, one-way ANOVA, Tukey’s post-hoc test).

#### 3.3.3. Thalamus

Phenotyping of dividing cells was performed in three thalamic nuclei: laterodorsal (LD), lateral posterior (LP), and ventral (V) (Figure 4 and Figure 5b). Similar to the assessment of EdU-labeled cell density, the thalamus exhibited the most substantial increase in the number of dividing cells one week after TBI, particularly in the laterodorsal and lateral posterior nuclei (LD—IC: 1 ± 0.2, Sh: 4 ± 1.5, Tr: 41 ± 10, *p* = 0.0003; LP—IC: 1 ± 0.3, Sh: 3 ± 1.6, Tr: 11 ± 1.7, *p* = 0.0002; V—IC: 3 ± 0.8, Sh: 8 ± 1.9, Tr: 30 ± 12.1, *p* = 0.0364, one-way ANOVA, Tukey’s post-hoc test) (Figure 4). We found that this strong proliferative response was caused by a dramatic increase in the number of EdU^+^/Nestin-GFP^−^/Iba^+^ microglia in all examined nuclei (LD—IC: 0 ± 0.1, Sh: 1 ± 0.3, Tr: 25 ± 7.8, *p* = 0.0023; LP—IC: 0 ± 0.2, Sh: 1 ± 0.5, Tr: 8 ± 2.6, *p* = 0.006; V—IC: 0 ± 0.2, Sh: 6 ± 1.4, Tr: 42 ± 16.9, *p* = 0.017, one-way ANOVA, Tukey’s post-hoc test). In addition, most of the newly formed microglia were found in the LD and LP nuclei, located closest to the lesion area and displaying a hypertrophied cell body shape. (Figure 5b). The number of EdU^+^/Nestin-GFP^−^/GFAP^+^ astrocytes was increased only in the lateral dorsal (IC: 0 ± 0.1, Sh: 0 ± 0.3, Tr: 6 ± 2.4, *p* = 0.0115, one-way ANOVA, Tukey’s post-hoc test) and lateral posterior nuclei (IC: 0 ± 0, Sh: 0 ± 0, Tr: 1 ± 0.4, *p* = 0.0092, one-way ANOVA, Tukey’s post-hoc test), but not in the ventral nucleus, which is furthest from the lesion area (IC: 0 ± 0, Sh: 1 ± 0.6, Tr: 3 ± 1.5, *p* = 0.1522, one-way ANOVA, Tukey’s post-hoc test) (Figure 4). We also found an increase in EdU^+^/Nestin-GFP^+^/GFAP^−^ cells in the lateral dorsal nucleus compared with the “Intact Control” group (IC: 1 ± 0.2, Sh: 3 ± 0.8, Tr: 5 ± 1.2, *p* = 0.0201, one-way ANOVA, Tukey’s post-hoc test), which was probably due to an increase in EdU^+^/Nestin-GFP^+^/Olig2^+^ cells; however, we found no significant difference in their number (IC: 1 ± 0.5, Sh: 2 ± 1.3, Tr: 5 ± 1.2, *p* = 0.1292, one-way ANOVA, Tukey’s post-hoc test) (Figure 4).

#### 3.3.4. Optic Tract

Phenotyping of EdU-labeled cells in the optic tract was performed at both 1 and 7 weeks post-impact. Unlike other structures, in the optic tract, we found no suppression in proliferative activity 7 weeks after impact; instead, an increase was observed (Figure 4). One week after LFPI, we were unable to identify the cell type responsible for the increase in the total number of EdU-labeled cells (Figure 4). In contrast, 7 weeks after TBI, we found an increase in the number of EdU^+^/Nestin-GFP^−^/GFAP^+^ astroglia (IC: 0 ± 0.1, Sh: 0 ± 0.2, Tr: 5 ± 0.9, *p* < 0.0001, one-way ANOVA, Tukey’s post-hoc test) and EdU^+^/Nestin-GFP^−^/Iba1^+^ microglia (IC: 0 ± 0.1, Sh: 0 ± 0.2, Tr: 6 ± 1.4, *p* < 0.0001, one-way ANOVA, Tukey’s post-hoc test) typical for the other structures (Figure 4). Meanwhile, we also found a significant increase in the number of EdU^+^/Nestin-GFP^+^/GFAP^+^ cells (IC: 0 ± 0.1, Sh: 1 ± 0.1, Tr: 2 ± 0.4, *p* = 0.0002, one-way ANOVA, Tukey’s post-hoc test) and EdU^+^/Nestin-GFP^+^/Olig2^+^ OPCs (IC: 2 ± 0.5, Sh: 1 ± 0.3, Tr: 5 ± 0.9, *p* = 0.0004, one-way ANOVA, Tukey’s post-hoc test) with the two control groups, which was not found in any of the structures described above (Figure 4 and Figure 5c).

In summary, we demonstrated that LFPI induced astrogliosis and even more intense microgliosis in all four subcortical structures, which were not directly affected by the TBI. In addition, we showed a significant increase in oligodendrocytes following TBI: EdU^+^/Nestin-GFP^−^/Olig2^+^ immature/mature oligodendrocytes in the striatum, and EdU^+^/Nestin-GFP^+^/Olig2^+^ OPCs in the substantia nigra.

### 3.4. LFPI Modified the Activation of GFAP-Expressing Astroglia and Iba1-Expressing Microglia in the Thalamus and Striatum

**Figure 6:**
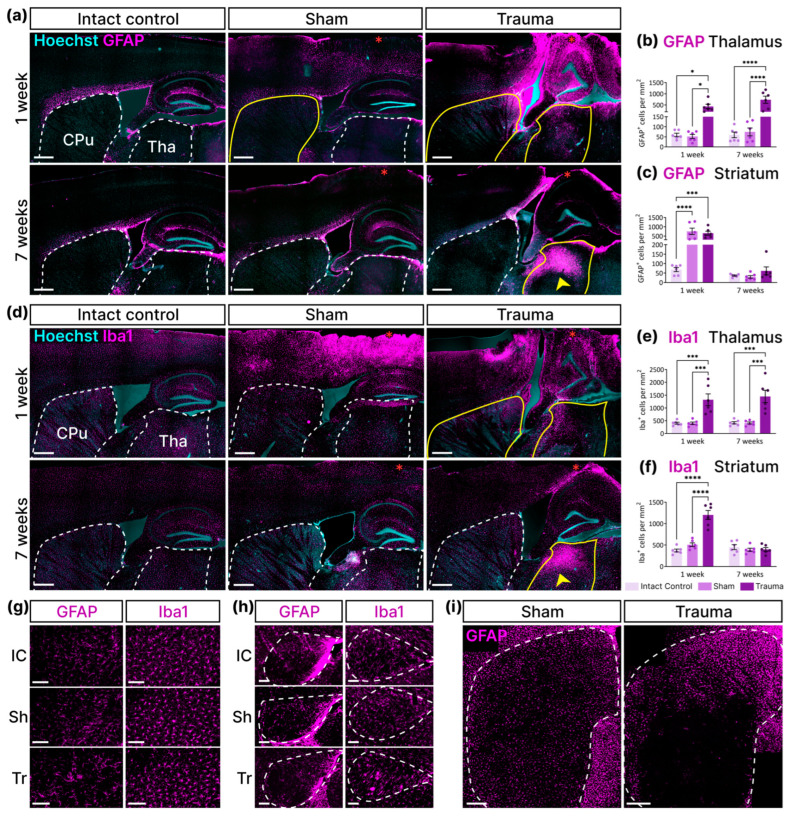
LFPI leads to different dynamics of total GFAP and Iba1 expression in the thalamus and striatum. (**a**,**d**) Representative images show the dynamics of GFAP expression in astrocytes (**a**) and Iba1 expression in microglia (**d**). Dotted lines indicate the thalamus (Tha) and striatum (CPu). Yellow solid lines show increased GFAP (**a**) and Iba1 (**d**) expression levels. Yellow arrowheads indicate local GFAP (**a**) and Iba1 (**d**) overexpression in the thalamus. Red asterisks indicate the lesion site. The scale bar is 500 mm. (**b**,**c**) Density of GFAP-labeled cells in the thalamus (**b**) and striatum (**c**). (**e**,**f**) Density of Iba1-labeled cells in the thalamus (**e**) and striatum (**f**). (**b**) Two-way ANOVA. Factor “Week”: *p* = 0.0968; factor “Impact”: *p* < 0.0001; interaction: *p* = 0.1113. (**c**) Two-way ANOVA. Factor “Week”: *p* < 0.0001; factor “Impact”: *p* = 0.0004; interaction: *p* = 0.0005. (**e**) Two-way ANOVA. Factor “Week”: *p* = 0.6545; factor “Impact”: *p* < 0.0001; interaction: *p* = 0.8939. (**f**) Two-way ANOVA. Factor “Week”: *p* < 0.0001; factor “Impact”: *p* < 0.0001; interaction: <0.0001. (**b**,**c**,**e**,**f**) Tukey’s post-hoc test. *—*p* < 0.05, ***—*p* < 0.001, ****—*p* < 0.0001. Data are presented as mean ± SEM (*n* = 6 for each group). (**g**,**h**) Representative images show no visible differences in the number of GFAP^+^ and Iba1^+^ cells in the substantia nigra (**g**) and optic tract (**h**) one week after LFPI. The dashed line indicates the optic tract. Abbreviations: IC—“Intact Control”, Sh—“Sham”, Tr—“Trauma”. The scale bar is 100 µm. (**i**) Representative images show the different distribution patterns of GFAP-positive cells in the striatum of the “Sham” and “Trauma” groups one week after TBI. The scale bar is 300 µm.

Since we showed that most dividing cells are microglia and astroglia, we analyzed the distribution of total GFAP- and Iba1-labeled cells to observe inflammation in dynamics and determine the fate of glial cells. We found no apparent changes in the number of GFAP- and Iba1-positive cells in the substantia nigra and optic tract of the 3 experimental groups (Figure 6g,h), in contrast to the striatum and thalamus, in which we quantified the density of GFAP- and Iba1-labeled cells at 1 and 7 weeks after injury (Figure 6a–f). We found that LFPI resulted in active astrogliosis and microgliosis in the thalamus 1 week after injury (Figure 6a,b,d,e; two-way ANOVA, factor “Impact”: *p* (GFAP) < 0.0001, *p* (Iba1) < 0.0001). Moreover, 7 weeks after impact, we found local overexpression of both GFAP and Iba1 in the dorsal thalamus (Figure 6a,d). However, no increase in the total density of GFAP- or Iba-labeled cells was found compared to the “Trauma” group 1 week after TBI (Figure 6b–e; two-way ANOVA; Tukey’s post-hoc test: *p* (GFAP) = 0.0923, *p* (Iba1) = 0.9861). In the striatum, we observed active astrogliosis 1 week after injury in mice of both the “Trauma” and the “Sham” groups (Figure 6a,c; two-way ANOVA, factor “Impact”: *p* (GFAP) = 0.0004). Moreover, sham surgery resulted in a more diffuse astroglia activation, whereas LFPI caused astrogliosis in the dorsal striatum (Figure 6i). An increase in Iba1 level was shown only in the “Trauma” group but not in the “Sham” group (Figure 6d,f). Seven weeks after the impact, we found a decrease in GFAP and Iba1 expression in the striatum compared to control levels (Figure 6a,c,d,f). Thus, we found that increased expression of GFAP and Iba1 was maintained in the thalamus even 7 weeks after LFPI. We also found differences in the expression of glial markers in the striatum. In contrast to microglia, the number of GFAP-activated astroglia was increased not only by traumatic brain injury but also by sham surgery.

### 3.5. The Number of Neural Precursor Cells in the Striatum Increased 7 Weeks After TBI

**Figure 7:**
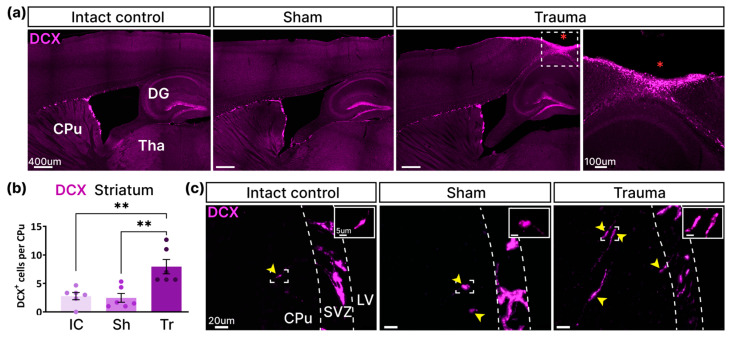
TBI induced the formation of new neural precursors in the striatum 7 weeks after injury near the SVZ. (**a**) Representative images show DCX expression in the regions close to the impact site. The red asterisk indicates the lesion site. The dotted box is represented at a larger magnification on the right. Abbreviations: DG—dentate gyrus, Tha—Thalamus, CPu—striatum. (**b**) The number of DCX-labeled cells in “Intact Control” (IC), “Sham” (Sh), and “Trauma” (Tr) groups. One-way ANOVA, Tukey’s post-hoc test. **—*p* < 0.01 (*n* = 6 for each group). (**c**) Representative images show DCX-positive cells in the striatum. The graph is signed CPu—striatum, SVZ—subventricular zone, LV—lateral ventricle. The white dotted line indicates the SVZ boundaries. Yellow arrowheads indicate DCX^+^ cell bodies.

To investigate the neuroregenerative potential of non-neurogenic brain regions, we used a marker of late neuronal differentiation stages—doublecortin (DCX) expressed in neuroblasts and immature neurons. For this purpose, we performed DCX-labeled cell counts in previously sampled brain structures 7 weeks after TBI. We detected DCX expression at the lesion site and an almost complete absence of DCX in surrounding structures (Figure 7a). No DCX-positive cells were detected in the substantia nigra, thalamus, and optic tract of the three experimental groups. However, DCX-positive cells were identified in the striatum, predominantly near the subventricular zone (SVZ) (Figure 7c). Moreover, the total number of DCX-labeled cells in the striatum was significantly increased in the “Trauma” group compared to the two control groups (IC: 3 ± 0.6, Sh: 2 ± 0.8, Tr: 8 ± 1.3, *p* = 0.0012, one-way ANOVA, Tukey’s post-hoc test) (Figure 7b). Thus, we found that LFPI induced the formation of new neuronal cells in the striatum 7 weeks after TBI.

## 4. Discussion

Several lines of evidence suggest that in the mammalian brain, neurogenesis can occur beyond the main neurogenic niches, either through local progenitors or neuroblasts migrating from the SVZ. Various traumatic exposures, including TBI, can induce these processes as potential brain self-repair mechanisms. In this study, we demonstrate that the brain initiates robust regenerative processes following LFPI. Even in the presence of active inflammatory responses, including astrocytosis and microgliosis, we were able to detect trauma-induced neurogenesis in the striatum. Furthermore, we also detected oligodendrogenesis in the striatum and optic tract, which is generally not anticipated as an inflammatory response to the injury.

We investigated cell division in brain structures outside the main neurogenic niches at 1 and 7 weeks post-LFPI. By examining the proliferative response and the differentiation pathways of dividing cells during both acute and delayed phases of neurodegeneration, we sought to understand damage in regions distant from the injury site and evaluate their potential for self-repair.

Our results revealed an extensive proliferative response in most examined structures one week post-injury, with cell division rates returning to control levels by seven weeks post-TBI in all regions except the optic tract. This cell proliferation pattern aligns with previous findings from main neurogenic niches, where cell division peaks within the first week post-injury before gradually returning to baseline levels [37,42,43,44]. The LFPI model of brain injury we employed causes diffuse nervous tissue damage and neurodegeneration across multiple brain structures [45,46], likely explaining the extensive inflammation-associated proliferative response. So far, enhanced cell divisions following LFPI outside the injured neocortex and main neurogenic niches have only been documented in the thalamus [47,48]. In contrast, studies using controlled cortical impact or traumatic axonal injury models have shown increased cell proliferation in the neocortex, striatum, thalamus, and hypothalamus [18,23]. These studies also suggest that the proliferative response is associated with astro- and microglia activation, likely indicating an inflammatory response to TBI. Notably, we found that sham surgery increased the number of EdU-labelled cells within the insular and piriform cortices distant from the lesion site at one week, suggesting that even without visible dural damage, craniotomy may cause mechanical trauma to the nervous tissue [49].

We found that at seven weeks post-LFPI, increased cell proliferation was found exclusively in the optic tract, with a continued increase in the number of dividing cells. Unlike other studied structures, the optic tract comprises a bundle of nerve fibers crucial for visual pathways [50]. Previous studies have documented optic tract degeneration within the first week and month after injury [51,52], along with nerve fiber demyelination one week after injury, micro- and astroglial activation persisting from first days up to seven months after the impact [51,52,53,54]. As the optic tract lies at the base of the skull opposite the actual lesion site, its damage could be caused by the contrecoup [55]. These findings suggest that, unlike the gray matter, white matter structures, such as the optic tract, exhibit a prolonged and even enhanced proliferative response, which persists over an extended period following traumatic injury.

Phenotypic analysis of EdU-labeled cells showed that in all selected structures showing robust proliferative response—striatum, substantia nigra, thalamus (laterodorsal and lateroposterior nuclei), and optic tract—the enhancement of cell divisions was primarily due to EdU^+^/Nestin-GFP^−^/GFAP^+^ astroglial cells and EdU^+^/Nestin-GFP^−^/Iba1^+^ cells microglial cells. These findings align with the results of other TBI models one week following injury, where a similar response was related to the inflammation and glial scar formation [56]. The response confirms the transformation of astro- and microglia into activated forms, characterized by hypertrophied cell bodies and thickened cell branches. In addition, we showed that one week after LFPI, the number of Iba1-labeled cells correlates with the number of EdU^+^ cells across all experimental groups; notably, we found a significant increase in GFAP-labeled cells in the sham-operated animals. These results may indicate that both markers’ accumulation has a unique activation threshold, with GFAP showing higher sensitivity.

Seven weeks post-injury, despite no increase in dividing cells in the thalamus, we observed a substantial accumulation of Iba1 and GFAP in its dorsal region, where peak proliferative response was observed one week post-LFPI. Thalamic cell division activation was often observed after TBI, highlighting this region’s vulnerability to damage [4,19,48,57], likely due to thalamocortical white matter tract damage [58], leading to secondary structural damage. The distribution pattern of Iba1 and GFAP may indicate the formation of a persistent astroglial scar in the thalamus and its long-term dysfunction.

We demonstrated an increase in EdU^+^/Nestin-GFP^+^/GFAP^+^ cells in the striatum and substantia nigra at one week post-impact and in the optic tract at seven weeks. Nestin, an intermediate filament expressed early in brain development, serves as a marker of neural progenitor cells (NPCs). In the SGZ, cells co-expressing Nestin and GFAP are identified as quiescent neural progenitors (QNPs) [39]. During neuronal differentiation, these cells lose GFAP expression while temporarily maintaining Nestin expression. Conversely, in the astrocyte differentiation pathway, Nestin expression is lost, while GFAP persists through astrocyte maturation [59]. However, this classification does not apply outside the SGZ. It was suggested that increased co-expression of GFAP and Nestin in the cortex following focal ischemia might indicate reversion to an immature cell state, potentially reflecting astrocyte reprogramming towards neuronal differentiation [60,61]. Alternatively, Nestin induction in astroglia may involve cytoskeleton remodeling during astrocyte hypertrophy of astrocytes following traumatic injury [62] and is not associated with neuronal differentiation.

Beyond neurogenesis, nervous tissue recovery depends on oligodendrocytes, which maintain nerve fiber conductivity. We demonstrated the formation of new OPCs (EdU^+^/Nestin-GFP^−^/Olig2^+^ cells) across studied structures in both intact and injured brains. In the adult brain, these cells almost invariably differentiate into mature myelinating oligodendrocytes, with potential for neuronal differentiation, but never develop astrocytes [63], indicating ongoing oligodendrogenesis throughout the lifespan. Remarkably, almost all Nestin-GFP-labeled progenitor cells in the analyzed structures were OPCs, although, in the adult brain, they were typically present as neuronal precursors, astrocytes, and pericytes [59].

Our studies revealed an increase in EdU^+^/Nestin-GFP^−^/Olig2^+^ cells in the striatum, representing immature or mature oligodendrocytes. To our knowledge, this presents the first quantitative analysis of striatal oligodendrogenesis following TBI, although new oligodendrocyte formation has been previously documented in damaged cortex [15]. Oligodendrogenesis in the adult brain remains less studied compared to embryonic development [63]. While this process likely serves to remyelinate damaged striatal axons, its therapeutic value for brain repair requires further investigation.

We also found an increase in the number of EdU^+^/Nestin-GFP^+^/Olig2^+^ OPCs in the optic tract 7 weeks after LFPI, consistent with findings in other white matter regions up to three months post-injury [64,65]. While evidence suggests that optic nerve axon remyelination primarily occurs through pre-existing oligodendrocytes [66], our results indicate the possible involvement of newborn cells in repairing damaged retinal axons.

We did not detect initial stages of neuronal progenitor differentiation 1-week post-LFPI, which might indicate neurogenic potential in the examined brain structures. Quantitative analysis of DCX-labeled cells at 7 weeks post-injury revealed their presence only in the striatum, suggesting an absence of neurogenic potential in the substantia nigra, thalamus, and optic tract (the latter being white matter).

Our results describe for the first time neurogenesis in the striatum after TBI. Most of the available reports about neurogenesis in the striatum after focal brain damage are related to rodent ischemic stroke models [67,68,69,70]. The BrdU-labeled cells in the ipsilateral to trauma striatum were observed in a CCI model in the first post-injury days [13,71]. Our observation of an increased number of striatal DCX-labeled cells following injury also aligns with findings from other pathologies [72,73,74,75]. The proximity of neuronal progenitors to the SVZ may indicate their migration from this neurogenic niche [67,68,69,70,74,76,77,78], although local neural progenitor pools may also exist [12].

TBI-induced changes represent an intricated continuum of pathological and compensatory/adaptive changes across all brain levels, from molecular and cellular to neuronal networks. Our findings highlight LFPI-induced changes from the perspective of endogenous brain regenerative capacity supporting post-trauma recovery. One week post-injury, selected brain regions believed to be non-neurogenic exhibited considerable transient proliferative response of glial cells, subserving neuroinflammatory events. Trauma-induced striatal oligodendrogenesis and neurogenesis may indicate self-repair mechanisms resembling those reported in focal ischemia models. Animal stroke models demonstrate enhanced striatal neurogenic potential as a post-ischemic self-repair mechanism.

Our notion that neurogenesis occurs in the striatum is supported by human studies, which suggest that new neurons can be formed in the adult striatum (see [79] for review). Although endogenous progenitor cells may provide limited striatal neuronal regeneration, the functional relevance of striatal neurogenesis remains obscure, unlike the well-documented migration of ischemia-induced neurons and neuroblasts from the subventricular neurogenic niche to the striatum [67]. Note, however, that trauma-induced neurogenesis may potentially lead to the formation of aberrant neuronal networks underlying brain pathologies, as observed in epileptogenesis [80]. Definitive interpretation of our findings requires evidence linking the discovered cellular events to functional aspects of post-trauma recovery.

Our study was conducted exclusively on sexually mature male mice, which imposes some limitations on interpreting the data obtained, particularly their direct transfer to females. TBI is known to induce aromatase expression in astrocytes, a key enzyme involved in estradiol (E2) synthesis [81]. E2, through its nuclear (ERs) and membrane (GPER1) receptors, activates intracellular pathways leading to enhanced neurogenesis and neuroprotection. Therefore, a study of cell divisions after TBI in females is necessary to interpret our results definitively.

## 5. Conclusions

Our results indicate that LFPI triggers extensive proliferative responses beyond main neurogenic niches, primarily through astro- and microglial activation as part of the inflammatory response. Furthermore, we demonstrated oligodendrogenesis in the striatum and optic tract, suggesting potential self-repair of nerve fibers by remyelination. In addition, evidence of post-TBI neurogenesis detected only in the striatum indicates possible neurogenic potential specific to this brain region. Further investigation of the ‘self-repair’ potential of subcortical regions after TBI and the molecular mechanisms underlying the observed changes is necessary to develop interventions, such as growth factors or stem cell therapy, to enhance recovery processes after trauma.

## Data Availability

Data are contained within the article.
